# Novel Multiparametric Nomogram for Overall Survival Prediction in Complicated Intra-Abdominal Infection: A Multicenter Study in China

**DOI:** 10.3389/fmed.2021.627416

**Published:** 2021-02-22

**Authors:** Sisi Huang, Limin Chen, Jiao Liu, Sheng Zhang, Lidi Zhang, Zhenliang Wen, Yizhu Chen, Dechang Chen

**Affiliations:** ^1^Department of Critical Care Medicine, Ruijin Hospital, Shanghai Jiao Tong University School of Medicine, Shanghai, China; ^2^Department of Critical Care Medicine, Ruijin Hospital North, Shanghai Jiao Tong University School of Medicine, Shanghai, China

**Keywords:** nomogram, complicated intra-abdominal infection, prognosis, APACHE II, SOFA

## Abstract

**Background:** Complicated intra-abdominal infections (cIAIs) in the abdominal cavity or within an abdominal organ are numerous and frequent dangerous entities in the treatment of critically ill patients. Early clinical evaluation is necessary.

**Methods:** This retrospective multicenter study included patients from 10 intensive care units (ICUs). Risk factors for the overall survival (OS) of patients with cIAI were selected using least absolute shrinkage and selection operator regression, and a nomogram was constructed subsequently. Calibration curve and receiver operating characteristic (ROC) curve were used to evaluate the calibration and discriminative ability.

**Results:** In total, 544 patients diagnosed with cIAI were enrolled and divided into the study (*n* = 276) and validation (*n* = 268) sets. Sex, acute gastrointestinal injury, acute kidney injury, rare bacterium infection, Charlson score, and APACHE II score were identified as independent risk factors and were constructed for the nomogram. The nomogram showed marked calibration capability with a concordance index (C-index) of 0.909 and 0.831 in the study and validation set, respectively. Compared with the common clinical prognostic scoring system, the nomogram achieved the highest discrimination ability with an area under the curve (AUC) value of 0.91 and 0.83 in the study set and validation set, respectively.

**Conclusions:** Our newly constructed nomogram provides a useful tool for risk stratification and prognosis evaluation of cIAI.

## Summary

A multiparameter nomogram that especially included acute gastrointestinal injury (AGI) for complicated intra-abdominal infection (cIAI) prognosis evaluation was the first to be established. Sex, AGI, AKI, rare bacterium infection, Charlson score, and APACHE II score were identified as the risk factors. Compared with the commonly used scoring system in ICU, SOFA, and APACHE II, the nomogram presented better overall net benefits in decision curve analysis (DCA) and higher area under the curve (AUC) value in operating characteristic (ROC) curve.

## Introduction

Intra-abdominal infections (IAIs) are responsible for nearly 20% of sepsis cases and are the second most common cause of infectious morbidity and mortality after pneumonia in intensive care units (ICUs) (1). IAIs are further classified as uncomplicated and complicated. Complicated intra-abdominal infections (cIAI) are more likely to cause drug-resistant bacterium infections, surrounding organ damage, and even systemic inflammatory reactions, subsequently contributing to the accumulation of hospitalization costs, length of stay, and morbidity (2, 3). Mortality associated with cIAI is generally high at 23–38% (4, 5). Achieving prompt control over infection of an anatomic source is the cornerstone of cIAI management but is not always successful (4).

Several risk factors including delayed interventions, antibiotic-resistant pathogens, high severity of illness, advanced age, poor nutritional status, and pre-existing chronic medical conditions have been reported to cause treatment failure (6–9). Early clinical evaluation is essential for the illness stratification and the subsequent decision-making process, and even for auditing and research. However, specific scoring system is unavailable. Oddeke (10) also reported that “none of the widely-used scoring systems to predict overall outcome in critically ill patients are of clinical value.” Nevertheless, few studies have comprehensively explored the risk factors for cIAI prognosis.

Therefore, this study aimed to identify factors that significantly influence the mortality of patients with cIAI in the ICU. Three different general organ function scores including the Charlson, Acute Physiologic and Chronic Health Evaluation II (APACHE II), and Sequential Organ Failure Assessment (SOFA) scores as well as several organ specific evaluation systems like the Glasgow Coma Scale (GCS), Chinese DIC scoring system (CDSS), acute kidney injury (AKI), and acute gastrointestinal injury (AGI) were investigated. We also incorporated patient characteristics, comorbidities, and infection source for a comprehensive assessment. The first nomogram for cIAI prognosis was constructed and confirmed in this study.

## Methods

### Study Population

This was a retrospective, multicenter study conducted in 10 hospitals including Ruijin Hospital North, the First Affiliated Hospital of Wenzhou Medical School, The Second Affiliated Hospital of Zhejiang University School of Medicine, the First Hospital of Lanzhou University, the First People's Hospital of Kunshan, Huashan Hospital, Changhai Hospital, Minhang Hospital, Qingpu Branch of Zhongshan Hospital, and the Seventh People's Hospital of Shanghai University of Traditional Chinese Medicine from January 2017 to October 2018. In total, 544 patients (age, 18–80 years) who were diagnosed with cIAI were enrolled in this study. Patients with primary peritonitis, with missing clinical data, or whose hospital stay was shorter than 48 h were excluded. This retrospective study was reviewed and approved by the Ruijin Hospital North. The included patients were randomly divided into a study set (*n* = 276) and validation set (*n* = 268) at a ratio of 1:1.

### Date Collection

The patient characteristics and clinical data of each patient were carefully collected and scrutinized. Clinical data such as the Charlson score, APACHE II score, SOFA score, GCS score, DIC score, AGI Grade, AKI Grade, and liver function were acquired on the first day in the ICU. Variables related to intra-abdominal infection included infection sites such as the biliary system, pancreas, and intestine, abdominal trauma, spontaneous peritonitis, and others. Pathogens having the highest drug resistance during hospitalization were recorded. Pathogens were classified into Gram-positive bacteria (*Enterococcus* spp., *Streptococcus* spp.), Gram-negative bacteria (*Escherichia coli, Klebsiella, Acinetobacter baumannii, Pseudomonas aeruginosa*, and *Enterobacter* spp.), rare bacterium infection (see *Definitions*), and fungi. Comorbidities occurring before admission included stroke, chronic obstructive pulmonary disease (COPD), chronic liver disease (CLD), diabetes, chronic kidney disease (CKD), malignancy, and hemopathy. The day of discharge or death was considered as the end point of the study.

### Definitions

Complicated intra-abdominal infection (cIAI) was defined as a generalized inflammatory process extending beyond the hollow viscus of origin into the peritoneal cavity that affects multiple organs and causes abscesses or peritonitis (11). Rare bacterium infection was defined as seldom-seen bacterium infection of cIAI such as *Proteus* spp., *Serratia* spp., *Staphylococcus* spp., and *Stenotrophomonas maltophilia*. Multidrug-resistant (MDR) bacteria refer to the bacteria that are resistant to three or more kind of commonly used antibiotics, including extensive drug resistance (XDR) and pan-drug resistance (PDR).

### Construction and Validation of the Nomogram

We incorporated all the clinical data as prognostic features to select the most useful predictive variables in the study group. The least absolute shrinkage and selection operator (LASSO) regression with 10-fold cross-validation was used to shrink all the regression coefficients toward zero. The penalty parameter lambda controls the amount of shrinkage, so lambda. min [the Lambda at which the minimal MSE (Mean Square Error) is achieved] was identified at first, and lambda. 1sd (one standard deviation of lambda. min) was used to select features for the nomogram construction of cIAI overall survival (OS).

A calibration curve was used to assess consistency between the nomogram-predicted survival probability and the actual fraction survival probability. According to the median risk probability of death predicted by the nomogram, patients with cIAI were classified into high- and low-risk groups. The potential association of the nomogram score with OS was first assessed in the study cohort and was then validated in the validation cohorts using Kaplan–Meier survival analysis. The clinical utility of the nomogram model was assessed by a decision curve analysis (DCA) in the testing and independent validation cohorts by quantifying the net benefits at different threshold probabilities. The receiver operating characteristic (ROC) curve and area under the curve (AUC), which is useful to estimate the predictive accuracy of prognostic predictors, were also used to assess and compare the performance of the nomogram and conventional evaluation systems such as APACHE II score, and SOFA score. A larger AUC indicated more accurate prognostic stratification.

### Statistical Analysis

Continuous variables not following normal distribution were expressed as median [interquartile range (IQR)] and analyzed using the rank-sum test. Categorical variables were expressed as frequency or ratio and were analyzed using the χ^2^ test. All statistical analyses were performed using R (version 3.6.2). The “glmnet” package was used to perform the LASSO Cox regression model analysis. All statistical tests were two-sided, and *P*-values < 0.05 were considered statistically significant.

## Results

### Clinical Characteristics

**Figure 1:**
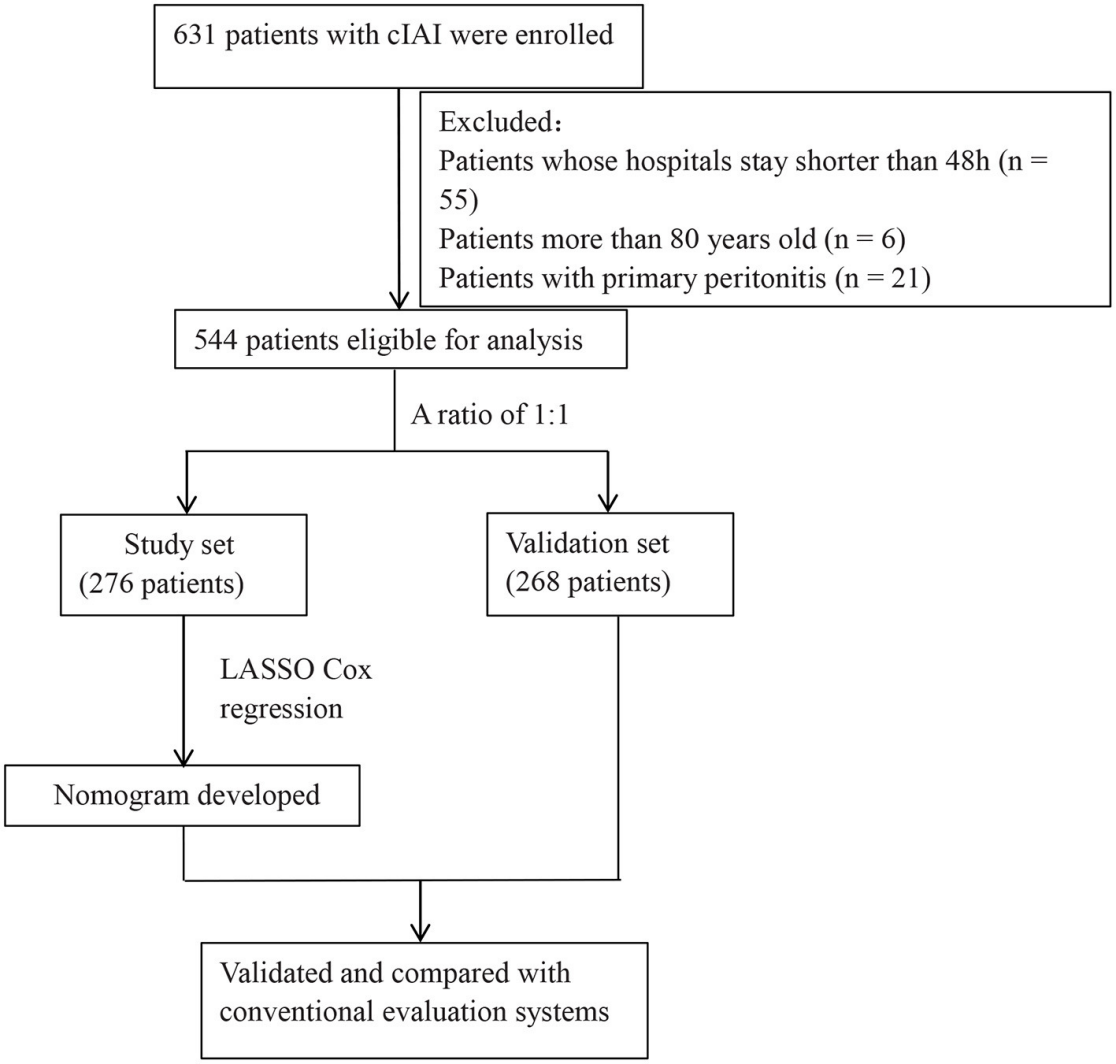
Flow chart of the present study.

Of 631 patients who was confirmed with cIAI, 544 patients were eligible for analysis (Figure 1). The clinical characteristics of the study and validation cohorts are shown in Table 1. The study and validation groups included 268 and 276 patients, respectively. There were 137 (51%) patients in the study set and 149 (54%) patients in the validation set that also had sepsis. The hospital duration of the study set was 18 (IQR, 11–30) days and the ICU duration was 9 (IQR, 4–17) days. The hospital duration of validation set was 18 (IQR, 12–34) days and the ICU duration was 8 (IQR, 4–16) days. The mortality in the study and validation set was 20% and 18%, respectively. All characteristics were well-balanced in both the study and validation cohorts (*p* > 0.05).

**Table 1:** Demographic and clinical characteristics of study patients.

**Variables**	**All**	**Study (*n* = 268)**	**Validation (*n* = 276)**	***p***
Age, Median (IQR), years	65 (53, 76)	65 (51, 75)	64.5 (55, 76)	0.467
Sex, Male, *n* (%)	355 (65.7)	180 (67.2)	175 (63.4)	0.406
**Comorbidities**				
Cardiovascular disease, *n* (%)	153 (28.1)	65 (24.3)	88 (31.9)	0.060
Dementia, *n* (%)	38 (7.0)	17 (6.3)	21 (7.6)	0.681
COPD, *n* (%)	28 (5.1)	15 (5.6)	13 (4.7)	0.784
CLD, *n* (%)	58 (10.7)	30 (11.2)	28 (10.1)	0.797
Diabetes mellitus, *n* (%)	86 (15.8)	46 (17.2)	40 (14.5)	0.462
CKD, *n* (%)	34 (6.3)	18 (6.7)	16 (5.8)	0.790
Solid tumor, *n* (%)	150 (27.6)	64 (23.9)	86 (31.2)	0.071
Hematological malignancies, *n* (%)	6 (1.1)	1 (0.4)	5 (1.8)	0.216
**Etiology**				
Biliary tract disease, *n* (%)	59 (10.85)	35 (13.1)	24 (8.7)	0.203
Acute pancreatitis, *n* (%)	69 (12.7)	30 (11.2)	39 (14.1)	0.203
Intestinal perforation or obstruction, *n* (%)	286 (52.6)	131 (48.9)	155 (56.2)	0.203
Abdominal trauma, *n* (%)	46 (8.5)	24 (9.0)	22 (8.0)	0.203
Complicated appendicitis, *n* (%)	39 (7.2)	21 (7.8)	18 (6.5)	0.203
Others, *n* (%)	45 (8.3)	27 (10.1)	18 (6.5)	0.203
**Microbioorganisms**				
Gram-Positive Bacteria				
*Enterococcus* spp., *n* (%)	80 (14.7)	38 (14.2)	42 (15.2)	0.825
*Streptococcus* spp., *n* (%)	10 (1.8)	3 (1.1)	7 (2.5)	0.34
Gram-Negative Bacteria				
*Klebsiella* spp., *n* (%)	37 (6.8)	17 (6.3)	20 (7.2)	0.804
*Escherichia coli, n* (%)	290 (53.3)	139 (51.9)	151 (54.7)	0.563
*Pseudomonas aeruginosa, n* (%)	22 (4.0)	10 (3.7)	12 (4.3)	0.883
*Acinetobacter baumannii, n* (%)	33 (6.1)	15 (5.6)	18 (6.5)	0.786
*Enterobacter* spp., *n* (%)	4 (0.7)	1 (0.4)	3 (1.1)	0.624
Rare bacterium infection, *n* (%)	65 (11.2)	26 (9.7)	35 (12.7)	0.334
Fungi, *n* (%)	35 (6.4)	19 (7.1)	16 (5.8)	0.660
MDR, *n* (%)	278 (51.1)	132 (49.3)	146 (52.9)	0.445
**Clinical Status at the Time of ICU Admission**				
Charlson score, Median (IQR)	1 (0, 3)	1 (0, 3)	2 (0, 3)	0.573
APACHE II score, Median (IQR)	11.5 (7, 18)	12 (7, 18)	11 (7, 18)	0.467
SOFA score, Median (IQR)	4 (1, 8)	4 (1, 8)	4.5 (1, 9)	0.676
GCS score, Median (IQR)	15 (14, 15)	14.5 (14, 15)	15 (14, 15)	0.324
CDSS score, Median (IQR)	3 (2, 5)	3 (2, 5)	3 (2, 5)	0.735
Liver injury, *n* (%)	222 (40.8)	114 (42.5)	108 (39.1)	0.471
Sepsis, *n* (%)	286 (52.6)	137 (51.1)	149 (54.0)	0.560
**AGI**				
No AGI, *n* (%)	89 (16.4)	51 (19.0)	38 (13.8)	0.316
AGI Grade1, *n* (%)	240 (44.1)	121 (45.1)	119 (43.1)	0.316
AGI Grade2, *n* (%)	101 (18.6)	45 (16.8)	56 (20.3)	0.316
AGI Grade3, *n* (%)	63 (11.6)	30 (11.2)	33 (12.0)	0.316
AGI Grade4, *n* (%)	51 (9.4)	21 (7.8)	30 (10.9)	0.316
**AKI**				
No AKI, *n* (%)	363 (66.7)	179 (66.8)	184 (66.7)	0.866
AKI Grade1, *n* (%)	62 (11.4)	30 (11.2)	32 (11.6)	0.866
AKI Grade2, *n* (%)	53 (9.7)	24 (9.0)	29 (10.5)	0.866
AKI Grade3, *n* (%)	66 (12.1)	35 (13.1)	31 (11.2)	0.866
**Treatment variables**				
Glucocorticoid, *n* (%)	94 (17.3)	42 (15.7)	52 (18.8)	0.388
CRRT, *n* (%)	67 (12.3)	33 (12.3)	34 (12.3)	1
Inappropriate antibiotic exposure, *n* (%)	27 (5.0)	10 (3.7)	17 (6.2)	0.269
**Outcomes**				
Length of hospitalization, Median (IQR), days	18 (11, 32)	18 (11, 30)	18 (12, 34)	0.272
ICU duration, Median (IQR), days	9 (4, 16)	9 (4, 17)	8 (4, 16)	0.929
In-hospital mortality, *n* (%)	103 (18.9)	53 (19.8)	50 (18.1)	0.700

*APACHE II, Acute Physiologic and Chronic Health Evaluation II; SOFA, Sequential Organ Failure Assessment; GCS, Glasgow Coma Scale; CDSS, Chinese DIC scoring system; AGI, acute gastrointestinal injury; AKI, acute kidney injury; CRRT, continuous renal replacement therapy; COPD, chronic obstructive pulmonary disease; CLD, chronic liver disease; CKD, chronic kidney disease; MDR, multidrug resistance; ICU, intensive care units*.

### Nomogram Development

**Figure 2:**
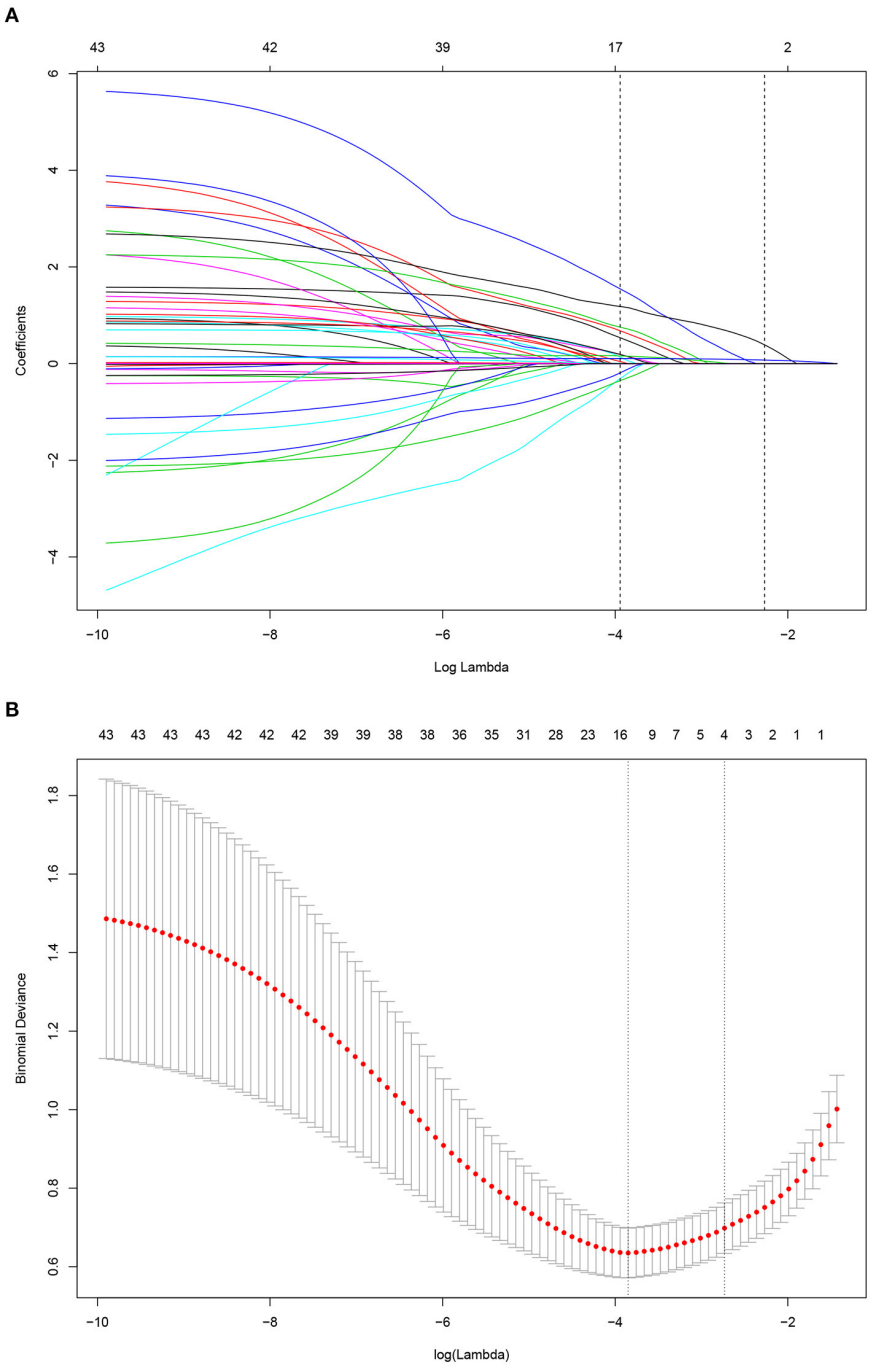
Risk factor selection using the least absolute shrinkage and selection operator (LASSO) Cox regression model. **(A)** LASSO coefficient profiles of the 35 factors for OS. **(B)** Tuning parameter (lamda) selection in the LASSO model used 10-fold cross-validation via minimum criteria for OS.

**Figure 3:**
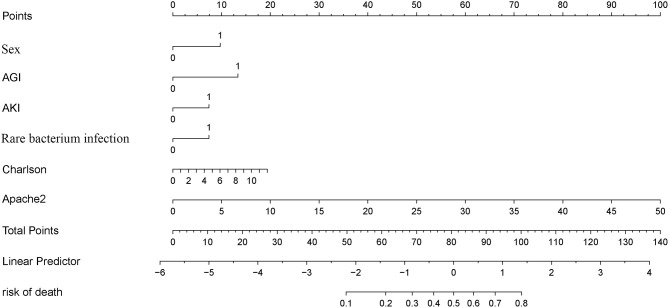
Nomogram of OS in complicated intra-abdominal infection.

A LASSO Cox regression model was used as a prognostic classifier, which successfully identified six potential predictors from the 37 features with non-zero coefficients in the study cohort (Figure 2). Sex, AGI, AKI, rare bacterium infection, Charlson score, and APACHE II score were independent risk factors (Figures 2, 3). A nomogram was constructed subsequently (Figure 3).

### Validation of Nomogram

**Figure 4:**
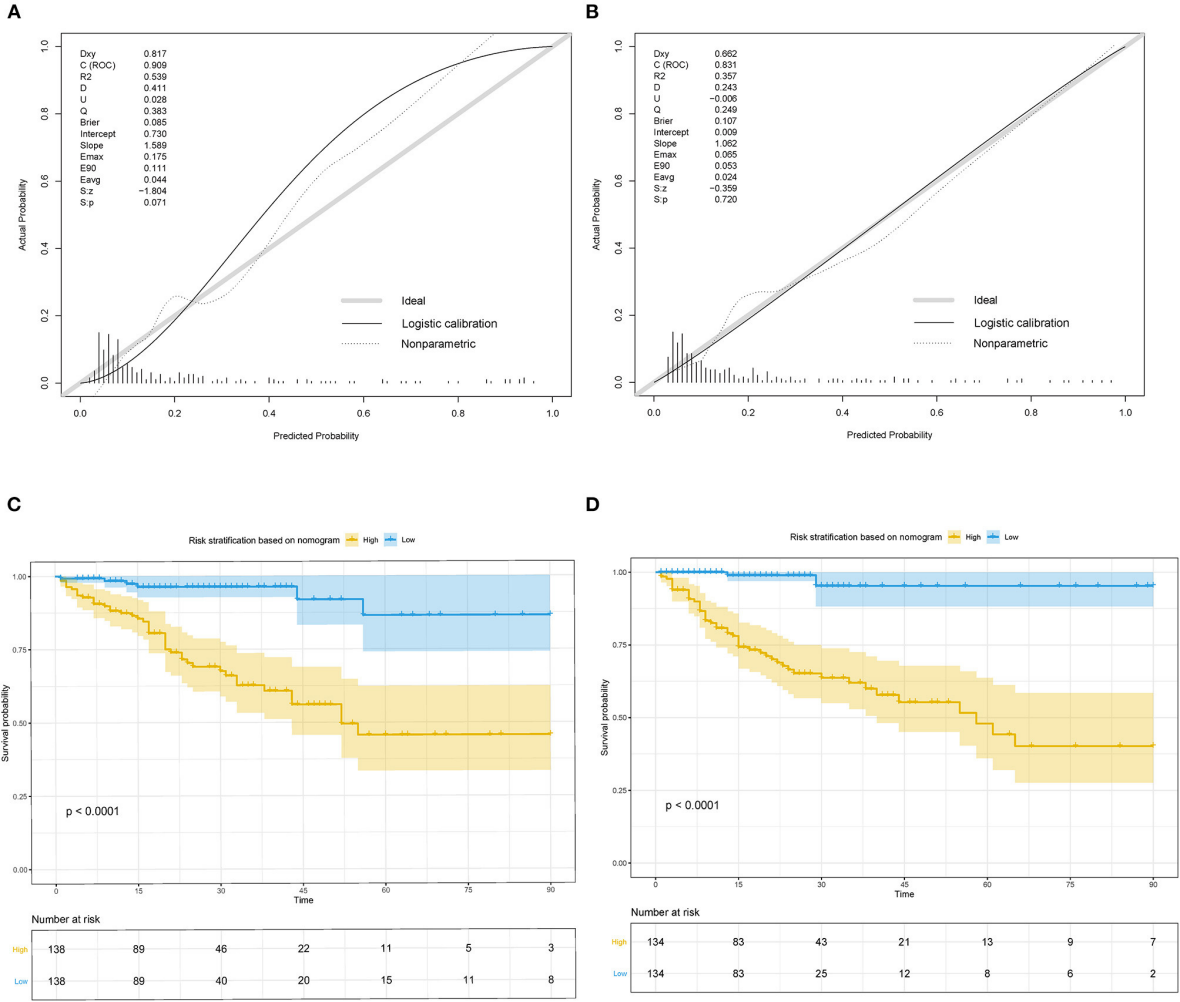
Calibration curves and Kaplan–Meier survival curves for the nomogram in study cohort and validation cohort of cIAI. **(A)** Calibration curve of the nomogram in the study cohort. **(B)** Calibration curve of the nomogram in validation cohort. **(C)** Kaplan–Meier survival curve of the nomogram in the study cohort. **(D)** Kaplan–Meier survival curve of the nomogram in the validation cohort.

The nomogram was well-calibrated as revealed by the calibration curves, and its prediction of death showed a good correlation between the actual observed outcome and the nomogram prediction (Figures 4A,B) in the study group (*p* > 0.05). This was further verified in the validation cohort (*p* > 0.05). The C-index of the nomogram for the prediction was 0.909 and 0.831 in the study and validation set, respectively. Accordingly, patients were classified into low-risk and high-risk groups based on the nomogram. Kaplan–Meier survival analysis showed that the actual survival rate of cIAI differed significantly from patients with low risk to those with high risk in both the study and validation sets (*p* < 0.001) (Figures 4C,D).

### Comparison of the Nomogram With Conventional Evaluation Systems

**Figure 5:**
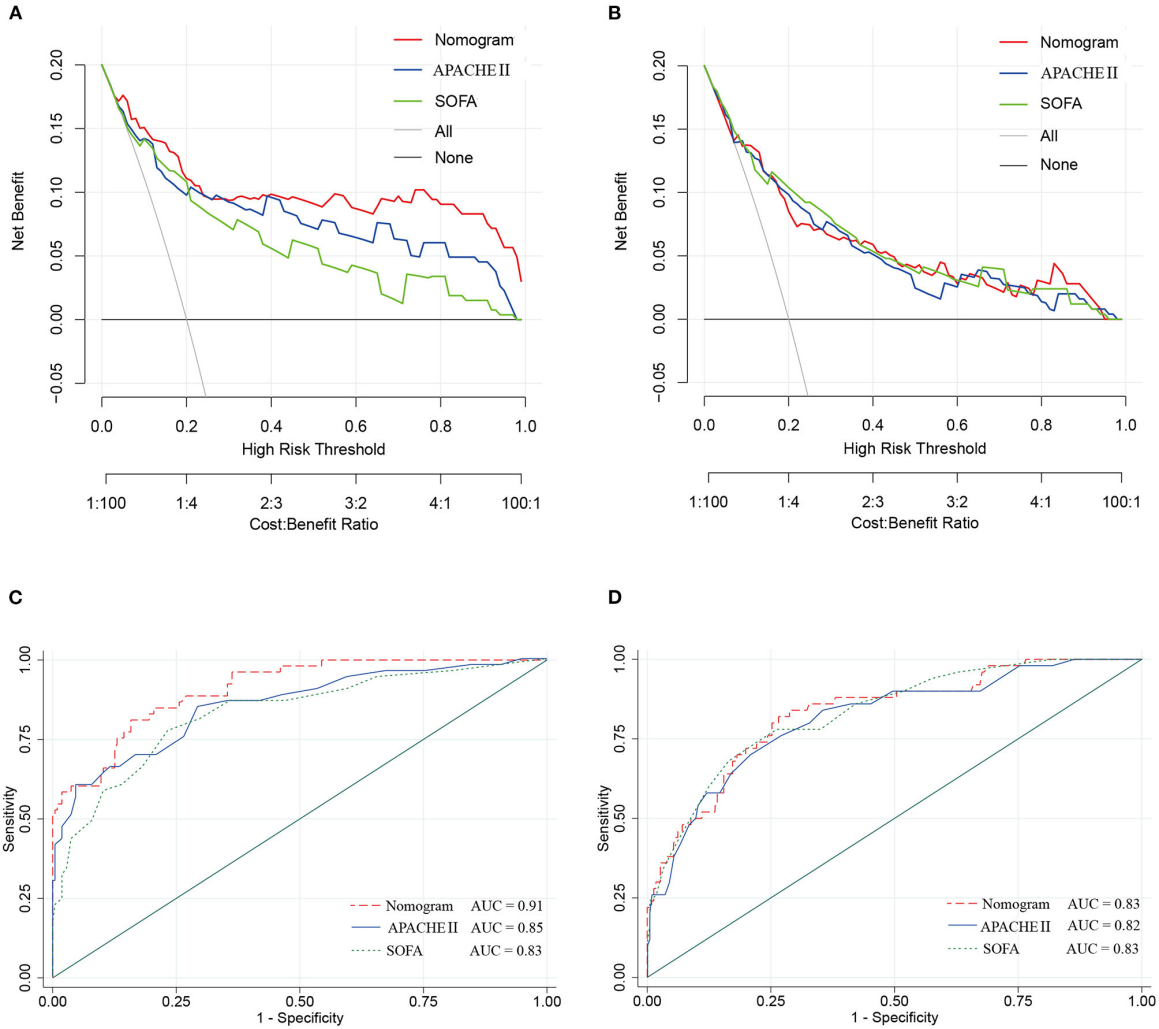
Decision curve and ROC curve for the nomogram in the study cohort and validation cohort of cIAI. **(A)** Decision curve of the nomogram in the study cohort. **(B)** Decision curve of the nomogram in the validation cohort. **(C)** ROC curve of the nomogram in the study cohort. **(D)** ROC curve of the nomogram in the validation cohort.

The DCA in the study cohort showed that our multiparametric nomogram had a better overall net benefit compared to the SOFA Score, APACHE II Score, and the treat-all patients strategy or the treat-none strategy at different threshold probabilities across the majority of the range between 4 and 100% (Figure 5A). DCA in the validation cohort showed an equal net benefit with the nomogram and the SOFA Score or APACHE II Score (Figure 5B).

The ROC curves (Figures 5C,D) were used to assess the discrimination ability of nomogram, SOFA, and APACHE II for the mortality of IAI. AUC values were the highest for the nomogram in the study group (AUC = 0.91) (Figure 5C), and the validation group (AUC = 0.83) (Figure 5D). The performances of SOFA and APACHE II are summarized in Table 2.

**Table 2:** AUC for Nomogram, APACHEII, and SOFA in the study cohort and validation cohort.

**Predictors**	**AUC**	**95% CI**	**Sensitivity** **(%)**	**Specificity** **(%)**	**Correctly** **classified**	**PPV**	**NPV**
**Study cohort**							
Nomogram	0.91	0.22–5.13	81.13	84.19	83.58%	80.00%	90.79%
APACHE II	0.85	0.21–2.90	84.91	70.70	73.51%	77.14%	88.84%
SOFA	0.83	0.30–3.33	77.36	76.74	76.87%	74.19%	87.34%
**Validation cohort**							
Nomogram	0.83	0.25–3.09	82.00	73.45	75.00%	66.67%	87.15%
APACHE II	0.82	0.38–3.37	70.00	79.20	77.54%	60.00%	86.06%
SOFA	0.83	0.30–2.99	78.00	73.89	74.64%	70.83%	86.90%

*AUC, area under curve; CI, confidence interval; APACHE II, Acute Physiologic and Chronic Health Evaluation II; SOFA, Sequential Organ Failure Assessment; PPV, positive predictive value; NPV, negative predictive value*.

## Discussion

We conducted a multicenter retrospective study in which we successfully enrolled 544 patients with cIAI to construct a nomogram for the evaluation of mortality risk. Sex, AGI, AKI, rare bacterium infection, Charlson score, and APACHE II score were identified as the risk factors, and were used to constitute the nomogram for prognosis prediction of cIAI in the study cohort. Internal validations further confirmed the nomogram as a successful prognostic evaluation system. Compared with the commonly used scoring system in ICU, SOFA, and APACHE II, the nomogram presented better overall net benefits in DCA and higher AUC value in ROC, which demonstrated the incremental value for evaluation of cIAI prognosis.

The Complicated Intra-Abdominal infection Observational Worldwide (CIAOW) study designed by the World Society of Emergency Surgery (WSES) had identified a critical clinical condition (severe sepsis and septic shock) upon hospital admission and was the most significant risk factor for death of cIAI (12). SOFA and APACHE II scores are commonly used in ICU for severity evaluation and are evaluated for the severity of cIAI (13–16). However, the prognosis prediction ability of APACHE II or SOFA score was controversial. Pascal et al. reported that APACHE II score was associated with the presence of *Pseudomonas aeruginosa* in peritoneal fluid culture but not with the prognosis (17). In a study of Kulkarni et al., APACHE-II score between 11 and 20 but not APACHE-II scores of 1 to 10 or >20 was shown to be a predictor of risk of mortality in patients with peritonitis due to hollow viscus perforation (18). Another study identified APACHE II ≥13 as the independent risk factors for failure of initial antibiotic therapy of cIAI (19). We identified APACHE II score but not SOFA score as the risk factor for patients with cIAI. According to our newly constructed nomogram, APACHE-II score between 11 and 20 achieved a point of 20 to 40 and APACHE II ≥13 achieved a point ≥26. The APACHE II score in the whole cohort ranged from 1 to 49 with a point range from 2 to 98 (Figure 3). We further compared the prognosis prediction ability of our newly constructed nomogram with APACHE II, SOFA score. Both DCA and ROC curves identified a better overall net benefit and better discrimination ability of the nomogram compared with APACHE II, SOFA score in the study group, even though the comparation of the three in the validation cohort showed an equal benefit.

Effects on function of specific organs, especially the gastrointestinal system, which was first and foremost affected, should be considered. The Working Group on Abdominal Problems of the European Society of Intensive Care Medicine firstly developed the definitions of AGI with four grades of severity, making it possible to estimate the gastrointestinal function of critically ill patients (20). AGI is widely used in ICU (21, 22). A multi-prospective study that recruited patients admitted to ICU diagnosed with AGI showed that AGI grading was positively correlated with all-cause mortality (23, 24). A retrospective study enrolled 286 critically ill patients with acute pancreatitis from ICU; the AGI grade distribution was 34.62% with grade 1, 22.03% with grade 2, 32.52% with grade 3, and 10.84% with grade 4, and the AGI grade was identified useful for predicting mortality (AUC = 0.854) (25). AGI grade upon ICU admission was firstly investigated for patients with cIAI in this study and was indicated as a risk factor of death. Patients with AGI got a point of 13 according to the nomogram (Figure 3). AKI is a common disease in the critically ill individuals, and is associated with high mortality (26). Alejandro's study showed that the incidence of AKI in surgical septic patients with secondary peritonitis was 58.8% (15). This study also had 181 (33.3%) patients with cIAI developed into AKI (Table 1) and identified AKI as an independent risk factor using the LASSO Cox regression model.

Risk factors such as old age, malignant disease, and pre-existing medical comorbidities may also attribute to the patient's underlying condition. Ana et al. (6) reported that elderly patients with intra-abdominal infection tend have a narrow therapeutic window, and old age is associated with significantly increased morbidity and mortality compared with younger patients. However, age was not identified in this study, which may be owing to the minimal differences between ages for all the patients had an advanced age [62.9 (61.5–64.3)] (Table 1). It is also indicated that cIAI mainly occurred in elderly patients. Interestingly, our study first identified sex as a risk factor, and male patients tended to have higher mortality and prolonged hospital stay. Similar observations were made by others in infectious diseases or septic shock, wherein males of any age showed worse prognosis (27). Females have better prognosis, and there is a hypotheses that it is probably ascribed to a higher neutrophilic inflammation and lower extracellular milieu's pH (28). Considering the comorbidities and malignant diseases, we chose Charlson score instead. Previous studies showed that Charlson score was significantly associated with all-cause mortality in patients with bacteremia (29) or sepsis (30), and our results corroborate these findings.

Achieving a prompt source control over the infection is crucial for abdominal infection management (4). Multi-pathogen infections often cause the failure of source control but are easily overlooked (31). This study identified infection with rare bacterium infection as the risk factor for cIAI prognosis. The underlying mechanism may be a lack of prompt and efficient antibacterial treatment when uncommon bacterium infection occurred. An emergent source control is necessary for cIAI with sepsis according to the most recent Surviving Sepsis Campaign Guidelines (32, 33). Antibiotic treatment mainly depends on experience, and cephalosporins and imipenem are commonly prescribed for cIAI (34). Furthermore, the infection rate of MDR bacteria was as high as 51.1% in this study, which was alarming.

The World Society of Emergency Surgery cIAIs Score Study (WISS) specifically constructed a WISS score, which includes severe sepsis or septic shock, healthcare-associated infections, delay in source control, origin of the IAIs, age, and immunosuppression to evaluate the severity of illness for patients with cIAI (35); it lacks verification and was principally used in surgical research since it was constructed in 2015 (36). Our newly constructed nomogram not only evaluated the whole condition (Charlson score and APACHE II score) but also included specific organ function (AGI, AKI) of patients with cIAI. Moreover, it emphasized the important role of timely specific pathogen identification for improving prognosis. The nomogram is more suitable for the evaluation of prognosis of cIAI in ICU. We even certificated the nomogram with a high calibration (Figures 4A,B) and discriminative ability (Figures 5C,D).

This study has a few limitations mainly related to its retrospective design. Firstly, the evaluation of infection control effect was not generally conducted in this study, which is different from the WISS score. However, as far as we know, infection control was more like a result after we evaluated the illness and took measures. Further studies will evaluate the factors influencing infection control effect. Secondly, owing to the limitation of data collection, we have not identified drug resistance of each pathogen but simply designated pathogens as MDR. It will affect the result of our evaluation; further study may identify more risk factors. Thirdly, the practicality of the nomogram was potentially limited due to the lack of external validation. Therefore, additional validation using datasets from other countries are encouraged.

## Conclusions

Our newly constructed nomogram, which included sex, AGI, AKI, rare bacterium infection, Charlson score, and APACHE II score, takes full consideration of the illness of cIAI, can predict its OS time accurately, and is considered a useful tool for risk stratification in cIAI.

## Data Availability Statement

The original contributions presented in the study are included in the article/Supplementary Material, further inquiries can be directed to the corresponding author/s.

## Ethics Statement

The studies involving human participants were reviewed and approved by Ruijin Hospital North. Written informed consent for participation was not required for this study in accordance with the national legislation and the institutional requirements.

## Author Contributions

All the authors have participated in clinical practice, literature retrieval, and viewpoint discussion in this article. SH and LC contributed to the writing of this article. SZ processed and analyzed the data. DC and JL revised this article. All authors read and approved the final manuscript.

## Conflict of Interest

The authors declare that the research was conducted in the absence of any commercial or financial relationships that could be construed as a potential conflict of interest.
