# Supplementary material for: Novel Multiparametric Nomogram for Overall Survival Prediction in Complicated Intra-Abdominal Infection: A Multicenter Study in China
Source: Front Med (Lausanne). 2021 Feb 22;8:627416. doi: 10.3389/fmed.2021.627416 (PMC7957962; doi:10.3389/fmed.2021.627416)
Supplement: Supplementary file 1 [file Table_1.DOC]

**1. APACHE II Score**

| Variable | Abnormal range | | | | | | | | | |
| --- | --- | --- | --- | --- | --- | --- | --- | --- | --- | --- |
|  | 0 (point) | 1 (point) | | | 2 (point) | | 3 (point) | | | 4 (point) |
| **Total acute physiology score** |  |  | | |  | |  | | |  |
| Temperature-rectal(℃) | 36.0-38.4 | 34.0-35.9  38.5-38.9 | | | 32.0-33.9 | | 30.0-31.9  39.0-40.9 | | | ≤20.9  ≥41.0 |
| MAP(mmHg) | 70-109 |  | | | 50-69  110-129 | | 130-159 | | | ≤49  ≥160 |
| HR(rpm) | 70-109 |  | | | 56-69  110-139 | | 40-54  140-179 | | | ≤39  ≥180 |
| RR(rpm)  (non-ventilated or ventilated) | 12-24 | 10-11  25-34 | | | 6-9 | | 35-49 | | | ≤5  ≥50 |
| PaO_2_(mmHg) | >9.33 | 8.13-9.33 | | |  | | 7.33-8.00 | | | <7.33 |
| A-aDO_2_(mmHg)  (FiO_2_ ≥ 0.5) | <26.67 |  | | | 26.67-46.53 | | 46.67-66.53 | | | ≤66.67 |
| Arterial PH | 7.33-7.49 | 7.50-7.59 | | | 7.25-7.32 | | 7.15-7.24  7.60-7.69 | | | <7.15  ≥7.70 |
| Serum sodium(mmol/L) | 130-149 | 150-154 | | | 120-129  155-159 | | 111-119  160-179 | | | ≤110  ≥180 |
| Serum potassium (mmol/L) | 3.5-5.4 | 3.0-3.4  5.5-5.9 | | | 2.5-2.9 | | 6.0-6.9 | | | <2.5  ≥7.0 |
| Serum creatinine (umol/L)  (Double point score for acute renal failure) | 53.04-123.76 |  | | | <53.04  132.6-167.96 | | 176.80-300.56 | | | ≥309.40 |
| Hematocrit (%) | 30.0-45.9 | 46.0-49.9 | | | 20.0-29.9  50.0-59.9 | |  | | | <20  ≥60 |
| WBC (total/mm3) | 3.0-14.9 | 15.0-19.9 | | | 1.0-2.9  20.0-39.9 | |  | | | <1.0  ≥40 |
| GCS score | =15 minus actual GCS | | | | | | | | | |
| **Age** | 44 | |  | | | 45-54 | | 55-64 | 65-74 (5 point)  75 (6 point) | |
| **Chronic health points*** | For elective postoperative patients (2 point) | | | For nonoperative or emergency postoperative patients (5 point) | | | | | | |

MAP, Mean arterial pressure; Rpm, revolutions per minute; HR, Heart reat; RR, Respiratory Rate; PaO_2_, arterial partial pressure of oxygen; FiO_2_, fraction of inspired oxygen; A -aDO_2_, alveolar arterial oxygen gradient; WBC, white blood count; GCS, glasgow coma scale; *If the patient has a history of severe organ system insufficiency or is immune-compromised assign points.

**2. Charlson score**

| Point |
| --- |
| Age |
| <50 years 0 |
| 50–59 years 1 |
| 60–69 years 2 |
| 70–79 years 3 |
| ≥80 years 4 |
| Myocardial infarction: History of definite or probable MI (EKG changes and/or enzyme changes) |
| No 0 |
| Yes 1 |
| CHF: Exertional or paroxysmal nocturnal dyspnea and has responded to digitalis, diuretics, or afterload reducing agents |
| No 0 |
| Yes 1 |
| Peripheral vascular disease: Intermittent claudication or past bypass for chronic arterial insufficiency, history of gangrene or acute arterial insufficiency, or untreated thoracic or abdominal aneurysm (≥6 cm) |
| No 0 |
| Yes 1 |
| CVA or TIA: History of a cerebrovascular accident with minor or no residua and transient ischemic attacks |
| No 0 |
| Yes 1 |
| Dementia (Chronic cognitive deficit) |
| No 0 |
| Yes 1 |
| COPD |
| No 0 |
| Yes 1 |
| Connective tissue disease |
| No 0 |
| Yes 1 |
| Peptic ulcer disease: Any history of treatment for ulcer disease or history of ulcer bleeding |
| No 0 |
| Yes 1 |
| Liver disease: Severe = cirrhosis and portal hypertension with variceal bleeding history, moderate = cirrhosis and portal hypertension but no variceal bleeding history, mild = chronic hepatitis (or cirrhosis without portal hypertension) |
| None 0 |
| Mild 1 |
| Moderate to severe 3 |
| Diabetes mellitus |
| None or diet-controlled 0 |
| Uncomplicated 1 |
| End-organ damage 2 |
| Hemiplegia |
| No 0 |
| Yes 2 |
| Moderate to severe CKD: Severe = on dialysis, status post kidney transplant, uremia, moderate = creatinine >3 mg/dL (0.27 mmol/L) |
| No 0 |
| Yes 2 |
| Solid tumor |
| None 0 |
| Localized 2 |
| Metastatic 6 |
| Leukemia |
| No 0 |
| Yes 2 |
| Lymphoma |
| No 0 |
| Yes 2 |
| AIDS |
| No 0 |
| Yes 6 |

**3. AGI Score**

AGI grade I (risk of developing GI dysfunction or failure)—the function of the GI tract is partially impaired, expressed as GI symptoms related to a known cause and perceived as transient.

AGI grade II (gastrointestinal dysfunction)—the GI tract is not able to perform digestion and absorption adequately to satisfy the nutrient and fluid requirements of the body. There are no changes in general condition of the patient related to GI problems.

AGI grade III (gastrointestinal failure)—loss of GI function, where restoration of GI function is not achieved despite interventions and the general condition is not improving.

AGI grade IV (gastrointestinal failure with severe impact on distant organ function)—AGI has progressed to become directly and immediately life-threatening, with worsening of MODS and shock.

As differentiation of the acute GI problem from previously existing chronic condition might be very difficult, we suggest using the same definitions also in cases where the condition (e.g. GI bleeding, diarrhoea, etc.) might be due to a chronic GI disease (e.g. Crohn’s disease). In patients on chronic parenteral feeding, GI failure (equal to AGI III) should be considered chronically present, and no new acute interventions to restore function are indicated. However, monitoring of IAH and exclusion of the new acute abdominal problems should be performed similarly as in AGI grade III management

**4.** AKI Stage

| Stage | Serum creatinine criteria | Urine output criteria |
| --- | --- | --- |
| 1 | Increase in serum creatinine of more than or equal to 0.3 mg/dl (≥ 26.4 μmol/l) or increase to more than or equal to 150% to 200% (1.5- to 2-fold) from baseline | Less than 0.5 ml/kg per hour for more than 6 hours |
| 2* | Increase in serum creatinine to more than 200% to 300% (> 2- to 3-fold) from baseline | Less than 0.5 ml/kg per hour for more than 12 hours |
| 3^#^ | Increase in serum creatinine to more than 300% (> 3-fold) from baseline (or serum creatinine of more than or equal to 4.0 mg/dl [≥ 354 μmol/l] with an acute increase of at least 0.5 mg/dl [44 μmol/l]) | Less than 0.3 ml/kg per hour for 24 hours or anuria for 12 hours |
| The staging system proposed is a highly sensitive interim staging system and is based on recent data indicating that a small change in serum creatinine influences outcome. Only one criterion (creatinine or urine output) has to be fulfilled to qualify for a stage.  * 200% to 300% increase = 2- to 3-fold increase. # Given wide variation in indications and timing of initiation of renal replacement therapy (RRT), individuals who receive RRT are considered to have met the criteria for stage 3 irrespective of the stage they are in at the time of RRT. | | |
